# Effects of superoxide anion attack on the lipoprotein HDL

**DOI:** 10.1007/s11010-022-04563-5

**Published:** 2022-10-11

**Authors:** Gaetana Napolitano, Gianluca Fasciolo, Maria Teresa Muscari Tomajoli, Alessandro Carlucci, Ester Ascione, Alfonso Salvatore

**Affiliations:** 1 Dipartimento di Scienze e Tecnologie, Università degli Studi di Napoli Parthenope, via Acton n. 38-I, 80133 Naples, Italy; 2 Dipartimento di Biologia, Università di Napoli Federico II, Complesso Universitario Monte Sant’Angelo, Via Cinthia, 80126 Naples, Italy; 3 S. Antimo Industrial Development Department, Kedrion Biopharma, Strada Statale 7 Bis 19, Sant’Antimo, 80029 Napoli, Italia; 4 International PhD Programme/UNESCO Chair “Environment, Resources and Sustainable Development”, Department of Science and Technology, Parthenope University of Naples, Naples, Italy

**Keywords:** HDL, Superoxide radical, Reverse cholesterol transport, APOA1, Lecithin cholesterol acyltransferase, Haptoglobin

## Abstract

High-density lipoprotein (HDL) is an anti-atherosclerotic lipoprotein. Thanks to the activity of apolipoprotein ApoA1, the principal protein component of HDL, this last is responsible for converting cholesterol into ester form and transporting excessive cholesterol to the liver (“reverse cholesterol transport” RCT). When HDL undergoes oxidation, it becomes dysfunctional and proatherogenic. ApoA1 is a target of oxidation, and its alteration affects RCT and contributes to atherosclerosis development. Until now, the mechanism of HDL oxidation is not fully understood and only hydroxyl radicals seem to induce direct oxidation of protein and lipidic components of lipoproteins. Here we demonstrate that superoxide radical, widely produced in early atherosclerosis, directly oxidizes HDL, and as a consequence, ApoA1 undergoes structural alterations impairing its anti-atherosclerotic functions. Our results highlight in an in vitro system the potential mechanism by which O_2_^·−^ triggers atherosclerotic pathogenesis in vivo. Our study gets the basis for therapeutic approaches focused on the management of superoxide generation in early atherosclerosis onset.

## Introduction

Atherosclerosis is a chronic and degenerative disease characterized by the accumulation of lipids and inflammatory cells in the arterial wall of blood vessels causing loss of elasticity, stiffening, and thickening mainly of large and medium-sized arteries [1]. Reactive oxygen species (ROS) play a pivotal role in the pathogenesis of atherosclerosis [2]. They include free radicals, such as superoxide (O_2_^−^) and hydroxyl radical (OH), and non-radical species, such as hydrogen peroxide (H_2_O_2_) and peroxynitrite (ONOOH). ROS are molecules extremely reactive, but in physiological conditions, the antioxidant defence systems counteract their production and maintain the oxidant–antioxidant balance [3]. When ROS production overwhelms the antioxidant defences of the cells, oxidative stress condition onset and biological molecules, such as lipids, proteins, and nucleic acids, are damaged [4]. Oxidative stress is crucial for the pathophysiology of atherosclerosis [5]. This condition induces intima injury and dysfunction with concomitant formation of oxidized low-density (LDL) and high-density (HDL) lipoproteins in circulation and lesion area.

The macrophages situated within the lesion area rapidly incorporate the oxidized LDL thanks to the greater affinity of lipoproteins for macrophage scavenger receptors, transforming themselves into foam cells. In turn, this leads to further plaque build-up in the arterial walls and the stimulation of inflammatory responses in the lesion [6]. Many factors, such as hypercholesterolemia, hypertension, diabetes, and smoke, are associated with the atherogenic process since they induce increased ROS production and systemic oxidative stress [6].

In contrast to low-density lipoprotein, high-density lipoprotein (HDL) is considered an anti-atherogenic particle capable of protecting the artery wall from the atherosclerotic process [7]. Atheroprotective functions of HDL include its anti-inflammatory, antioxidant, anti-apoptotic, anti-thrombotic, and vasodilatory properties [8]. ApoA1, the major protein component of HDL, is responsible for the anti-atherogenic property rather than the whole HDL particle. It plays a crucial regulative role in tissue cholesterol homeostasis by promoting a process known as “reverse cholesterol transport” (RCT), by which the excess cholesterol in peripheral tissues is removed and transported to the liver for catabolism [9]. At first, apoA1 initiates the efflux of phospholipids and cholesterol from the cell membrane, and in a second moment, it stimulates lecithin cholesterol acyltransferase (LCAT) for converting cholesterol into ester form [10]. ApoA1 also binds Haptoglobin (Hpt), a plasma glycoprotein that captures and transports to the liver the free Haemoglobin (Hb) to prevent Hb-triggered oxidative stress [11]. The role of Hpt in protecting ApoA1 from oxidative damage has been reported [11]. Hyka and co-workers [12] demonstrated the anti-inflammatory capacity of lipid-free apoA1 by inhibiting the release of IL-1 from monocytes/macrophages activated by contact with stimulated T lymphocytes. Furthermore, ApoA1 can lower the cellular levels of pro-IL-1 in LPS-primed mouse bone marrow-derived macrophages [13], reducing the inflammatory process.

When HDL undergoes oxidation, likely in chronic and acute inflammatory states, it loses the atheroprotective characteristics and becomes proatherogenic [14]. Multiple in vitro studies showed that different oxidants might readily modify HDL [8, 15]. It is reported that HDLs recovered from human atheroma are dysfunctional and extensively oxidized [16], and ApoA1 oxidized in Trp72 shows a potent pro-inflammatory activity ex vivo and an impaired capacity to stimulate HDL biogenesis in vivo [16].

However, the underlying mechanisms of oxidation of HDL remain unclear. When exposed to ROS, amino acids residues of apolipoproteins, such as arginine, lysine, proline, cysteine, threonine, leucine, and histidine, may undergo direct oxidation [17]. The oxidation of polyunsaturated fatty acids residues in lipoproteins may occur by two pathways: *i*) direct oxidation by ROS; *ii*) reactions with oxidized lipids [17]. To date, only hydroxyl radicals seem to induce direct oxidation of protein and lipidic components of lipoproteins [17].

In our study, we suggested that superoxide can be responsible for the direct oxidation of HDL and, as a consequence, the alteration of the structure and functionality of ApoA1. An increase in superoxide production characterizes the early stages of atherosclerosis and cardiovascular dysfunctions [18]. Several enzyme systems can produce superoxide in the vascular wall, such as NADPH oxidase, xanthine oxidase, enzymes of the mitochondrial respiratory chain, and dysfunctional endothelial NO synthase, which is responsible for the oxygen reduction uncoupled from NO [19].

Here we showed the ability of superoxide radicals to oxidize HDL (by electrophoresis and Western blotting). The oxidation affects also ApoA1. The following structural alterations of ApoA1 impair its capacity to bind Haptoglobin, evaluated by ELISA test, and mediate RCT, as revealed by LCAT assay.

## Experimental procedures

### Materials

Chemicals of the highest purity, human serum albumin (HSA), dextran sulphate (DS), cholesterol, cholesteryl linoleate, human Hpt (mixed phenotypes Hpt 1–2), 4-chloro-1-naphtol, 2,2’-azino-bis(3-ethylbenz-thiazoline-6-sulfonic acid) (ABTS), KO_2_, superoxide dismutase (SOD), catalase (CAT), and molecular weight markers were purchased from Sigma-Aldrich (St. Louis, MO, USA). KO_2_ was stored desiccated in the dark under anaerobic conditions. Human ApoA1 or HDL, and rabbit anti-human ApoA1 IgG were from Calbiochem (La Jolla, CA, USA). [1α, 2α-^3^H] Cholesterol (45 Ci/mmol) was obtained from Perkin Elmer (Boston, MA, USA). PVDF transfer membrane (Millipore, Bedford, MA, USA) were used. Sil-G plates for thin-layer chromatography (0.25 mm thickness) of Macherey–Nagel (Düren, Germany) were used. Commercial plasma samples were furnished by Kedrion Biopharma SpA (Barga, Lucca, Italy). Chelex 100 molecular biology grade resin was from BioRad laboratories.

### Methods

#### Oxidation of HDL

The oxidation system for HDL was constituted by a reaction mixture (TBS: 130 mM NaCl, 20 mM Tris–HCl, pH 7.4) with 50 or 100 µM of KO_2_ and 1 mg/mL lipoprotein. The incubation was performed for 15 min at 37 °C. Adding KO_2_ to an aqueous medium produces O_2_^−^ to oxidize HDL [20]. Radical production was monitored by measuring the absorbance at 734 nm of reaction mixtures supplemented with 450 µM of ABTS and maintained at 31 °C [21]. Basically, the reaction between O_2_^−^·and ABTS generates coloured ABTS^+^ cations characterized by an absorption peak at 734 nm [21]. In our preliminary experiments, the inclusion of SOD prevented the formation of ABTS^+^ cations. Furthermore, adding the H_2_O_2_ scavenger catalase did not affect the oxidation of ABTS. The KO_2_ concentration chosen for our experiments are similar to H_2_O_2_ concentration produced in pathological conditions [22]. KO_2_ stock solution was prepared according to Lokesh and Cunningham [20]. SOD (2800 units/mg) and (CAT) (2000 units/mg) were used to exclude that any oxygen metabolites linked to spontaneous dismutation of superoxide was responsible for the ABTS radical production.

Metals were removed from reaction mixture (prior to addition of KO_2_, lipoprotein, and ABTS) using Chelex 100 resin.

#### Electrophoresis and immunoblotting of oxidized HDL

Electrophoresis was carried out in denaturing and reducing conditions on 1.5-mm-thick slab gel, using the discontinuous system. The procedure for SDS-PAGE has been described in detail elsewhere [11] and samples containing 30–50 µg of proteins were used for the assay. After electrophoresis, the gels were fixed and stained according to Salvatore et al. [23]. Fixing and staining were omitted when the gel was processed for immunoblotting analysis.

Western blotting onto PVDF membrane and staining by antibodies were carried out essentially as previously described [23, 24]. After protein blotting, the membrane was rinsed in TBS, left for 15 min in 130 mM NaCl, 20 mM Tris (TBS) containing 0.05% (v/v) Tween 20 (T-TBS), and incubated with 1% (w/v) gelatine in T-TBS for 1 h at 37 °C. Then the membrane was incubated (1 h, 37 °C) with rabbit anti-Apo A1 IgG (1:200 in T-TBS). After treatment, the membrane was rinsed in T-TBS and incubated (1 h, 37 °C) with rabbit GAR-HRP IgG (1:300 in T-TBS). The membrane was rinsed again in T-TBS and finally immersed in TBS containing 0.03% H_2_O_2_ (v/v) and 4 mM 4-chloro-1-naphtol for the detection of immunocomplexes.

#### Hpt-ApoA1 binding capacity

The capacity of ApoA1 to bind Hpt was performed by ELISA as previously described [23]. In brief, microtitre plate wells were coated by incubation with 1 μg of antigen (native HDL or HDL previously oxidized with 10, 50, or 100 µM KO_2_) in 50 μl of a buffer containing 7 mM Na_2_CO_3_, 17 mm NaHCO_3_, and 1,5 mM NaN_3_ (pH 9.6). Wells were incubated with 55 μl of solution containing 0.5 µM Hpt (1 h at 37 °C). The primary antibody used for the method was an Anti-Hpt IgG diluted 1:1500 in T-TBS (pH 7.4) supplemented with 0.25% BSA. Sixty μl of GAR-HRP IgG diluted 1:3000 was used as secondary antibody to detect bound immunocomplexes by incubation for 1 h at 37 °C. Colour development was monitored at 492 nm as previously described [25].

#### LCAT assay

Samples of plasma furnished by Kedrion BioPharma and treated with 0.08% DS (50 kDa molecular mass) in 0.16 M CaCl_2_ to remove low-density and very low-density lipoprotein, were used as source of LCAT (DS-treated plasma) [25]. The enzyme activity was measured using a proteoliposome containing the apolipoprotein, phospholipids, and ^3^H-labelled cholesterol (ApoA1: lecithin:cholesterol = 1.5:200:18, molar contribution) as substrate, essentially according to published procedures [23]. The proteoliposomes oxidized for 1 h with 50 or 100 µM KO_2_ to obtain no more superoxide produced were used for the procedure.

The enzyme activity was expressed as Units (U) defining one unit of LCAT activity as nanomoles of cholesterol ester produced per hour per millilitre of plasma at 37 °C.

### Statistical analysis

The data, expressed as the means ± standard error of three different experiments, were analysed by the one-way or two-way variance analysis methods (*P* < 0.05), followed by Tukey post hoc test (Prism 9, GraphPad Software, LLC).

## Results

### ApoA1 oxidation by superoxide radicals

**Fig. 1:**
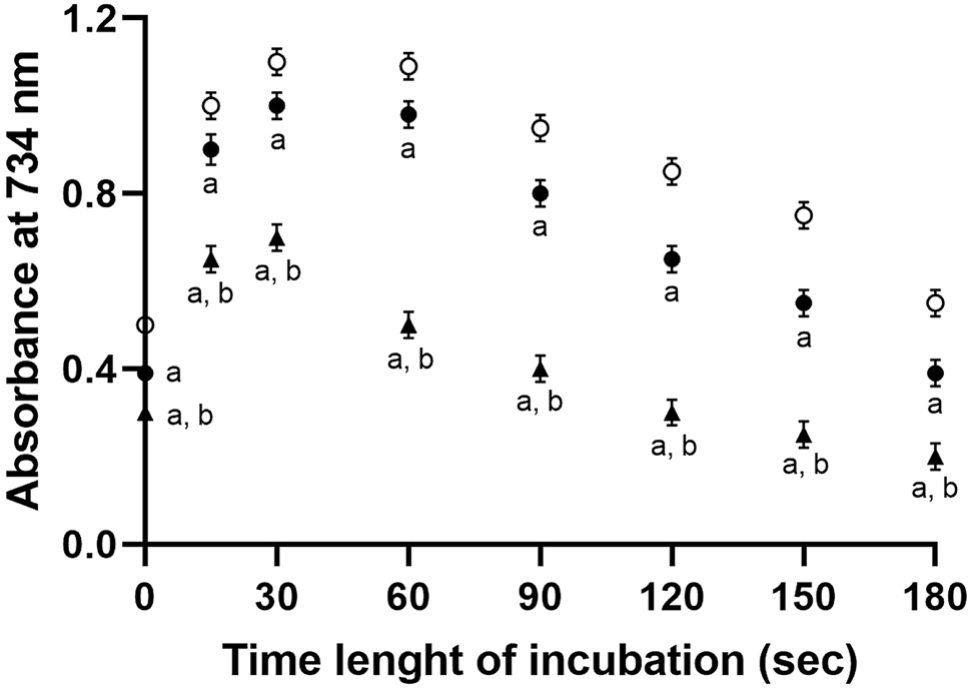
Oxidation of HDL. The incubation of a reaction mixture containing 450 mM ABTS with 50 or 100 µM KO_2_ produces superoxide radicals. The measure of the absorbance at 734 nm furnishes information concerning the formation and fading of the coloured ABTS^+^ cations after oxidation by superoxide radicals. Mixtures supplemented with HDL (1 mg/mL) were analysed. Open circles: 450 µM ABTS + 100 µM KO_2_. Filled circles: 450 µM ABTS + 50 µM KO_2_. Filled triangles: 450 µM ABTS + 50 µM KO_2_ + 1 mg/mL HDL. a significant respect data obtained by incubation of ABTS with 100 µM KO_2_. b significant respect data obtained by incubation of ABTS with 50 µM KO_2_. (One-way ANOVA, Tukey post hoc text, *P* < 0.001)

Our experiments showed that superoxide anions produced by adding KO_2_ to an aqueous medium can oxidize HDL. As reported in Fig. 1, the curve obtained with 450 mM ABTS and 100 µM KO_2_ (open circles) shows that the adduct level increased in less than 1 min from the reaction start, but the formed colour was not stable as observed in extended incubation. The use of lower KO_2_ concentration to oxidize ABTS (50 µM) induced a lower formation of the adduct (filled circles). This system of radical production allowed the oxidation of HDL. The addition of lipoproteins (1 mg/ml) to the reaction mixture induced the formation of coloured cations remarkably lower (triangles), indicating that both ABTS and HDL are substrates for oxidation by superoxide. The obtained data suggest that HDL undergoes O_2_^−^·oxidization by concentration like that of H_2_O_2_ produced in pathological conditions [21].

**Fig. 2:**
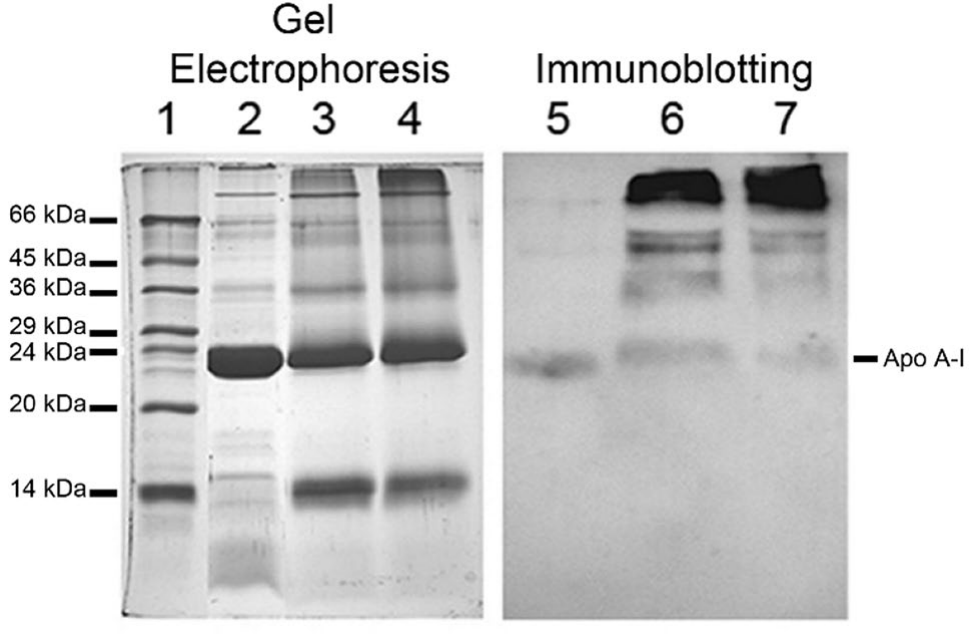
Electrophoresis and immunoblotting of oxidized HDL. The proteins were fractionated by SDS-PAGE and stained by Coomassie R250 or blotted onto PVDF membrane for immunostaining Lane 1: molecular weight standards, Coomassie staining. Lane 2: native HDL, Coomassie staining. Lane 3: HDL incubated with 50 µM KO_2_, Coomassie staining. Lane 4: HDL incubated with 100 µM KO_2_, Coomassie staining. Lane 5: native HDL, immunoblotting. Lane 6: HDL oxidized by 50 µM KO_2_, immunoblotting. Lane 7: HDL oxidized by 100 µm KO_2_, immunoblotting

The SDS-PAGE confirmed the oxidation of HDL (Fig. 2, lanes 2, 3, and 4). In comparison with the pattern of native HDL, the profile of oxidized HDL showed additional or more intense bands (lanes 3 and 4 versus lane 2), as demonstrated by the appearance of ~ 14 KDa bands. Moreover, the band corresponding to ApoA1 migration (MW 24 Kd), that we followed as marker of HDL oxidation, run broader for oxidized HDL than native one (apparent molecular weight slightly lower than 24 kd). The proteins separated by electrophoresis were also analysed by immunoblotting **(**Fig. 2, lanes 5, 6, and 7). In addition to native ApoA1, other protein bands characterized by a higher apparent molecular weight were detected (Fig. 2, lanes 6 and 7). These slow-migrating bands of Apo A1-like antigens might be a product of ApoA1 oxidation. Samples of oxidized HDL mostly contained a band more intense than from native HDL (Fig. 2, lane 6). Moreover, a slower band of comparable intensity was present (Fig. 2, lane 6). Probably minor oxidative modifications that do not influence the electrophoretic mobility are also present in ApoA1 of treated samples. In conclusion, the oxidation of HDL affects its electrophoretic mobility. Our data suggest that the proteins containing ApoA1 epitopes underwent an oxidation process triggered by superoxide radicals on HDL.

### Loss of function of Apo A1: effect on Hpt binding

**Fig. 3:**
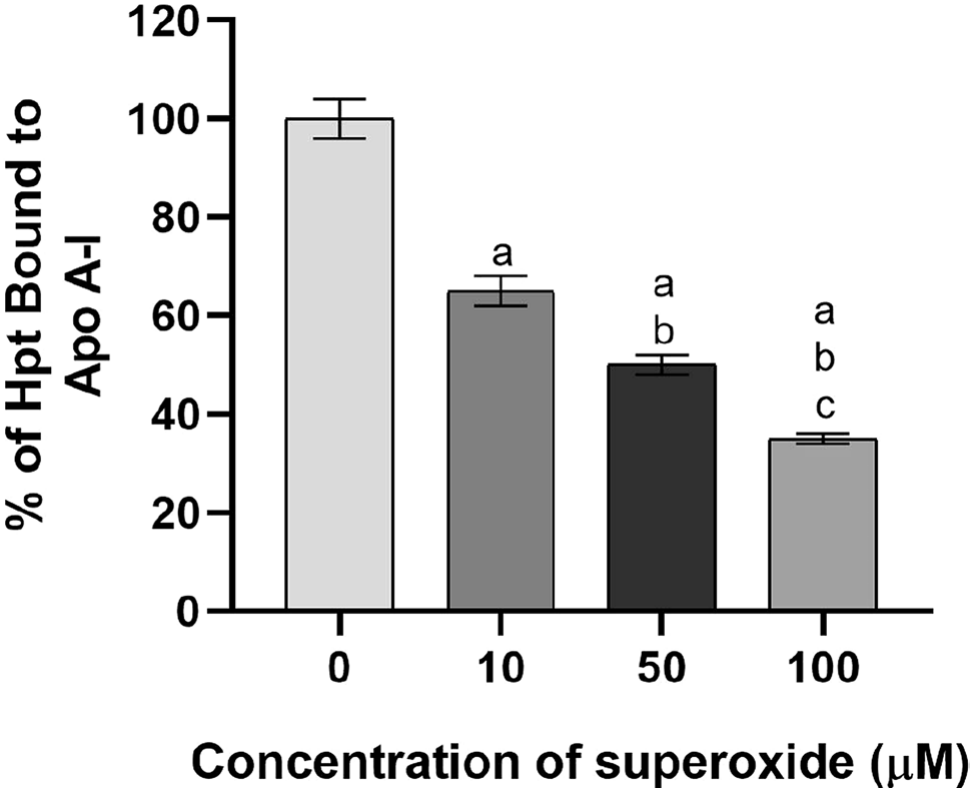
Hpt binding capacity of ApoA1. In the ELISA experiment, ApoA1 was oxidized in wells with 10, 50, or 100 µM KO_2_. Hpt binding dropped down to 65 (deep grey bar), 53 (black bar), and 31% (light grey bar), respectively. The data are reported as percent of the value obtained by incubation of Hpt alone (white bar on the left). The samples were analysed in triplicate: the data are expressed as means ± SD. a significant respect incubation of Hpt alone. b significant respect ApoA1 oxidized with 10 µM KO_2_. c significant respect ApoA1 oxidized with 50 µM KO_2_. (One-way ANOVA, Tukey post hoc text, *P* < 0.001)

The ApoA1 oxidation induces the loss of the capacity to bind Hpt. Figure 3 shows the percentage of Hpt bound to ApoA1 under different superoxide concentrations. Compared with native HDL, Hpt linked to ApoA1 dropped down with increasing concentration of superoxide. In particular, the decrease of the bond reaches 69% when a superoxide concentration of 100 μM is used.

### Loss of function of ApoA1: effect on stimulation of LCAT

**Fig. 4:**
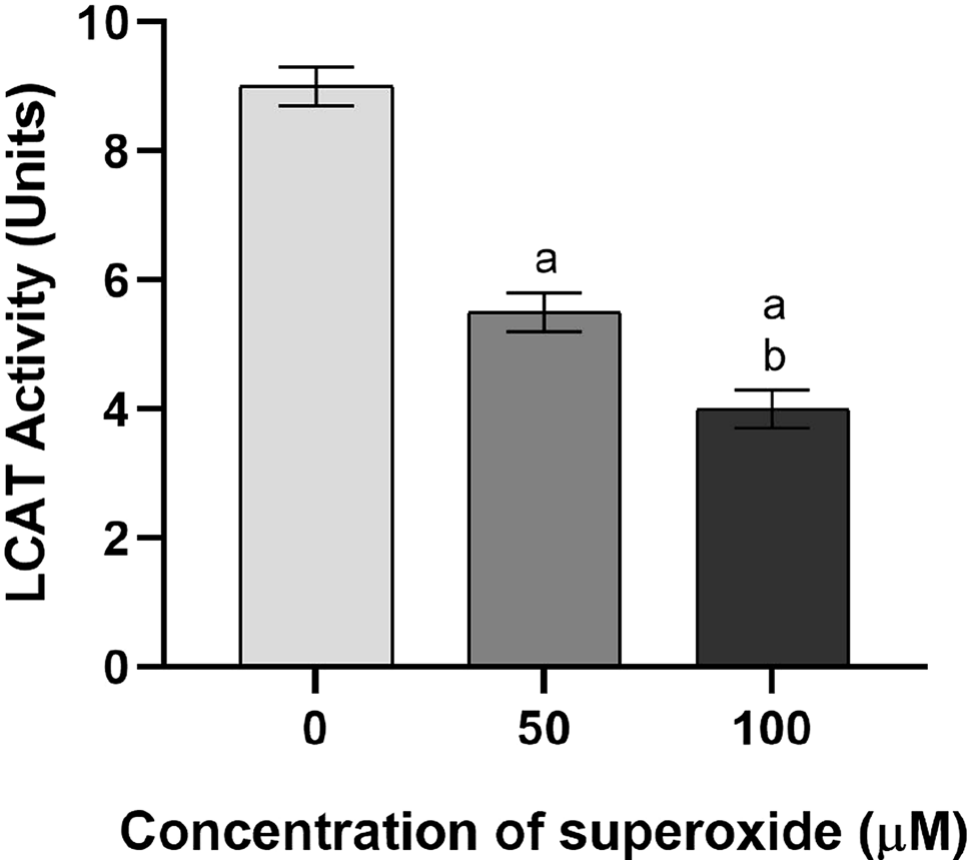
LCAT assay in the presence of oxidized proteoliposomes. DS-treated plasma was used LCAT source and the enzyme activity was assessed using a proteoliposome (ApoA1: lecithin: ^3^H-cholesterol, 1.5: 200: 18 molar ratio) as substrate. The proteoliposome was used as such (white bar) or after oxidation with 50 µM or 100 µM superoxide (deep grey bar and black bar, respectively). The samples were analysed in triplicate: the data are expressed as means ± SD. a significant respect proteoliposome not oxidized. b significant respect proteoliposome oxidized with 50 µM KO_2_ (One-way ANOVA, Tukey post hoc text, *P* < 0.001)

Oxidative damage of the ApoA1 structure might affect the protein capacity to stimulate the LCAT enzyme to the production of cholesteryl esters. Figure 4 shows LCAT activity under different superoxide concentrations.

Treatment without superoxide to validate ApoA1 stimulatory effect on LCAT is performed (0 µM). ApoA1-containing proteoliposomes stimulate the enzyme to esterify cholesterol (9.49 ± 0.16 U; *P* < 0.001). The oxidative treatment of proteoliposomes with 50 µM (5.66 ± 0.31 U, *P* < 0.003) and 100 µM (3.96 ± 0.31 U, *P* < 0.003) superoxide significantly impairs the stimulation of LCAT.

## Discussion

The results reported in this study highlighted for the first time a direct effect of superoxide on HDL. The competition between ABTS and HDL for superoxide anions generated from KO_2_ in an in vitro system [20, 21] supports this conclusion. The direct HDL oxidation by superoxide radicals is sustained by the preliminary observations in which the inclusion of SOD prior to the addition of KO_2_ prevented the formation of ABTS^+^ cations. Moreover, the oxidation of the ABTS was not influenced by adding the H_2_O_2_ scavenger catalase. These data together allow us to conclude that (i) O_2_^−^·is formed in aqueous solution by KO_2_ and is required for the oxidation of ABTS; (ii) the formation of ABTS^+^ cations is mediated by O_2_^−^·and not by other oxygen metabolites linked to spontaneous dismutation of superoxide. The production of superoxide anion occurs in several physio-pathological conditions [26], such as the atherosclerosis development [27]. Collin and co-workers showed that superoxide anion production is associated with early atherosclerosis conditions and cardiovascular dysfunctions in a rabbit model [18]. Different enzymes contribute to the increased production of vascular superoxide, such as NAD(P)H oxidases [28, 29] and both the endothelial and plasmatic Xanthine oxidase (XO) isoforms [30, 31]. The increased expression and activity of NAD(P)H oxidase subunits and xanthine oxidase in human atherosclerotic plaque sustain the involvement of superoxide in human coronary artery disease [32]. The endothelial nitric oxide synthase (eNOS) represents an additional source of superoxide [33]. In oxidative stress condition eNOS becomes uncoupled, and the enzyme no longer produces nitric oxide, that preserves the function of the endothelium [34], but superoxide [35]. Our results suggest that the increase of superoxide induces direct HDL oxidation. The finding of oxidized HDL in atherosclerotic lesions and the circulation of subjects with cardiovascular disease [8] support our data.

Exposure to superoxide widely impairs lipoprotein structure. The SDS-PAGE and Western blotting analysis performed on native and oxidized HDL using an antibody specific for ApoA1 apoprotein showed a protein structural change induced by oxidation. The finding that ApoA1 is a target of oxidative modification impairing its functions supports this result [36]. The myeloperoxidase enzyme, produced by macrophages within the atherosclerotic plaque in inflammation and oxidative stress condition, can modify in vitro several amino acid residues of human ApoA1 by producing nitrite and hypochlorite [15]. “Hot areas” for oxidation of ApoA1 were identified by tandem mass spectrometry with selective reaction monitoring mode [16]. Particularly, Tyr166 is a nitration site accounting for 8% of human atherosclerotic plaque, and its function is damaged compared with normal HDL [37]. The ApoA1 functionality is crucial in removing excess cholesterol from tissues and incorporating it into HDLs for reverse transport to the liver, getting the basis for atheroprotective events [8]. Under oxidation, ApoA1 loses its ability to mediate cholesterol efflux from cells [23], RCT becomes compromised, the cholesterol accumulates in the sub-intima cells of the vessel wall and triggers the atherosclerosis process [7]. As shown, the exposition of HDL to superoxide anions leads to the alteration of ApoA1 structure with the formation of high molecular weight protein aggregates. Many fragments formed adducts among themselves and/or with sequences from other HDL apolipoproteins or lipids. Some of these fragments maintained epitopes recognized by anti-ApoA1 antibodies. Other fragments of ApoA1 could be very short and probably not detected by the antibodies in our method. Unfortunately, our method does not allow to conclude that the superoxide anion is the direct responsible for the oxidation of ApoA1. The apoprotein could be directly oxidized by superoxide or indirectly by the lipids of HDL oxidized by the radical. It is reported that HDL is enriched in oxidized phospholipids in vivo in oxidative stress condition and these last can covalently modify apoproteins of HDL [14].

The loss of ApoA1 functions did not allow the protein to bind Hpt, a plasma glycoprotein, as revealed by the ELISA test. The primary biochemical role of Hpt is to sequester-free haemoglobin, thus protecting against iron-induced oxidative damage, inflammatory response, and cerebrovascular disease. It is also known to bind HDL on ApoA1 protein [38]. The Hpt might protect ApoA1 structure and function against hydroxyl radicals, as demonstrated by the non-formation of covalent adducts of this apolipoprotein with other proteins [39]. The inability of ApoA1 to bind Hpt contributes to its further oxidative damage. The structure alteration of ApoA1 does not allow the protein to mediate the HDL biogenesis [40]. In this process, the stimulation of ApoA1 on the enzyme LCAT, which catalyse the esterification of cholesterol molecules in growing HDL, is crucial [40]. LCAT activation by ApoA1 involves conformational changes in LCAT that stabilize its lid in an open state more competent to bind substrates [41]. It is reported that LCAT activity is lower or absent if the lipoprotein is missing or damaged [42]. Our LCAT assay demonstrated that ApoA1 did not efficiently stimulate the enzyme whether exposed to superoxide. Literature reporting that HDL biogenesis and catabolism affections can lead to dyslipidaemia [43] supports this result. This observation confirms the indirect link between RTC and superoxide levels. Experimental research performed on a primate model affected by moderately severe atherosclerosis and regression of atherosclerosis supports this link [44]. In this study, the remission of atherosclerosis relies on a reduction in vascular O_2_^−^·and NAD(P)H oxidase levels, thus contributing to the improvement in vasomotor function [44]. According to the authors, the reduction in vascular oxidative stress, and the subsequent stabilization of atherosclerotic lesions, may explain the association between the decrease in plasma cholesterol in patients with coronary atherosclerosis and major coronary events [45, 46].

## Conclusions and future perspectives

In conclusion, our findings suggest an important mechanism that contributes to the loss of HDL’s atheroprotective function. We demonstrated, for the first time, that the superoxide anion has a direct oxidative effect on HDL. This oxidative modification also affects native architecture and functions of ApoA1 so contributing to the alteration of RCT. In this context, the oxidation caused by superoxide can play an important role by contributing significantly to the development and progression of atherosclerosis. Our results allow us to plan a potential future therapeutic strategy for atherosclerosis management. Therapies aimed to tackle local superoxide generation at the site of atherogenesis (i.e. modulation of NAD(P)H oxidases, xanthine oxidase, eNOS activation) could be particularly useful in the early stages of the disease. The further combination with other potential antioxidant drugs could reveal a successful strategy to block the atherosclerosis onset and contain its progression. Further studies performed in in vivo experiments are requested for this purpose.

## Data Availability

The data that support the findings of this study are available on request from the corresponding author. The data are not publicly available due to privacy or ethical restrictions.

## Abbreviations

ApoA1: Apolipoprotein A1
LCAT: Lecithin cholesterol acyltransferase
HDL: High-density lipoprotein
RCT: Reverse cholesterol transport
Hpt: Haptoglobin
ROS: Reactive oxygen species
ABTS: 2,2’-Azino-bis (3-ethylbenz-thiazoline-6-sulfonic acid)

## References

[1] Hansson GK, Hermansson A (2011). The immune system in atherosclerosis. Nat Immunol.

[2] Goncharov NV, Avdonin PV, Nadeev AD, Zharkikh IL, Jenkins RO (2015). Reactive oxygen species in pathogenesis of atherosclerosis. Curr Pharm Des.

[3] Napolitano G, Fasciolo G, Venditti P (2021). Mitochondrial management of reactive oxygen species. Antioxidants.

[4] Venditti P, Napolitano G, Di Meo S (2015). Role of mitochondria and other ROS sources in hyperthyroidism-linked oxidative stress. Immunol Endocr Metab Agents Med Chem.

[5] Kattoor AJ, Pothineni NVK, Palagiri D, Mehta JL (2017). Oxidative stress in atherosclerosis. Curr Atheroscler Rep.

[6] Stocker R, Keaney JF (2004). Role of oxidative modifications in atherosclerosis. Physiol Rev.

[7] Xepapadaki E, Zvintzou E, Kalogeropoulou C, Filou S, Kypreos KE (2020). Τhe antioxidant function of HDL in atherosclerosis. Angiology.

[8] Rosenson RS, Brewer HB, Ansell BJ, Barter P, Chapman MJ, Heinecke JW, Kontush A, Tall AR, Webb NR (2016). Dysfunctional HDL and atherosclerotic cardiovascular disease. Nat Rev Cardiol.

[9] Sviridov D, Nestel P (2002). Dynamics of reverse cholesterol transport: protection against atherosclerosis. Atherosclerosis.

[10] Sorci-Thomas MG, Thomas MJ (2002). The effects of altered apolipoprotein A-I structure on plasma HDL concentration. Trends Cardiovasc Med.

[11] Salvatore A, Cigliano L, Bucci EM, Corpillo D, Velasco S, Carlucci A, Pedone C, Abrescia P (2007). Haptoglobin binding to apolipoprotein A-I prevents damage from hydroxyl radicals on its stimulatory activity of the enzyme lecithin-cholesterol acyltransferase. Biochemistry.

[12] Hyka N, Dayer JM, Modoux C, Kohno T, Edwards CK, RouxLombard P, Burger D (2001). Apolipoprotein A-I inhibits the production of interleukin-1 and tumor necrosis factor- by blocking contact-mediated activation of monocytes by T lymphocytes. Blood.

[13] Witkowski A, Carta S, Lu R, Yokoyama S, Rubartelli A, Cavigiolio G (2019). Oxidation of methionine residues in human apolipoprotein A-I generates a potent pro-inflammatory molecule. J Biol Chem.

[14] Gao D, Podrez EA (2018). Characterization of covalent modifications of HDL apoproteins by endogenous oxidized phospholipids. Free Radic Biol Med.

[15] Qin S (2020). LDL and HDL oxidative modification and atherosclerosis. Adv Exp Med Biol.

[16] Huang Y, Didonato JA, Levison BS, Schmitt D, Li L, Wu Y (2014). An abundant dysfunctional apolipoprotein A1 in human atheroma. Nat Med.

[17] Arai H, Kato Y (2014). Oxidative modification of lipoproteins. Lipid hydroperoxide-derived modification of biomolecules, subcellular biochemistry.

[18] Collin B, Busseuil D, Zeller M, Perrin C, Barthez O, Duvillard L (2007). Increased superoxide anion production is associated with early atherosclerosis and cardiovascular dysfunctions in a rabbit model. Mol Cell Biochem.

[19] Li H, Horke S, Förstermann U (2014). Vascular oxidative stress, nitric oxide and atherosclerosis. Atherosclerosis.

[20] Lokesh BR, Cunningham ML (1986). Further studies on the formation of oxygen radicals by potassium superoxide in aqueous medium for biochemical investigations. Toxicol Lett.

[21] Miller NJ, Rice-Evans C, Davies MJ, Gopinathan V, Milner A (1993). A novel method for measuring antioxidant capacity and its application to monitoring the antioxidant status in premature neonates. Clin Sci.

[22] Halliwell B, Gutteridge JMC (2000). Free radicals in biology and medicine.

[23] Salvatore A, Cigliano L, Carlucci A, Bucci EM, Abrescia P (2009). Haptoglobin binds apolipoprotein E and influences cholesterol esterification in the cerebrospinal fluid. J Neurochem.

[24] Venditti P, Napolitano G, Barone D, Pervito E, Di Meo S (2016). Vitamin E-enriched diet reduces adaptive responses to training determining respiratory capacity and redox homeostasis in rat heart. Free Radic Res.

[25] Spagnuolo MS, Cigliano L, D’Andrea LD, Pedone C, Abrescia P (2005). Assignment of the binding site for haptoglobin on apolipoprotein A-I. J Biol Chem.

[26] Brand MD (2020). Riding the tiger—physiological and pathological effects of superoxide and hydrogen peroxide generated in the mitochondrial matrix. Crit Rev Biochem Mol Biol.

[27] Poznyak AV, Grechko AV, Orekhova VA, Chegodaev YS, Wu WK, Orekhov AN (2020). Oxidative stress and antioxidants in atherosclerosis development and treatment. Biology.

[28] Griendling KK, Sorescu D, Ushio-Fukai M (2000). NAD(P)H oxidase: role in cardiovascular biology and disease. Circ Res.

[29] Sorescu D, Szocs K, Griendling KK (2001). NAD(P)H oxidases and their relevance to atherosclerosis. Trends Cardiovasc Med.

[30] Ohara Y, Peterson TE, Harrison DG (1993). Hypercholesterolemia increases endothelial superoxide anion production. J Clin Invest.

[31] White CR, Darley-Usmar V, Berrington WR, McAdams M, Gore JZ, Thompson JA (1996). Circulating plasma xanthine oxidase contributes to vascular dysfunction in hypercholesterolemic rabbits. Proc Natl Acad Sci USA.

[32] Guzik TJ, Sadowski J, Guzik B, Jopek A, Kapelak B, Przybylowski P (2006). Coronary artery superoxide production and nox isoform expression in human coronary artery disease. Arterioscler Thromb Vasc Biol.

[33] Heitzer T, Krohn K, Albers S, Meinertz T (2000). Tetrahydrobiopterin improves endothelium-dependent vasodilation by increasing nitric oxide activity in patients with type II diabetes mellitus. Diabetologia.

[34] Li H, Forstermann U (2009). Prevention of atherosclerosis by interference with the vascular nitric oxide system. Curr Pharm Des.

[35] Li H, Forstermann U (2013). Uncoupling of endothelial NO synthase in atherosclerosis and vascular disease. Curr Opin Pharmacol.

[36] Martínez-López D, Camafeita E, Cedó L, Roldan-Montero R, Jorge I, García-Marqués F (2019). APOA1 oxidation is associated to dysfunctional high-density lipoproteins in human abdominal aortic aneurysm. EBioMedicine.

[37] Di Donato JA, Aulak K, Huang Y, Wagner M, Gerstenecker G, Topbas C (2014). Site-specific nitration of apolipoprotein A-I at tyrosine 166 is both abundant within human atherosclerotic plaque and dysfunctional. J Biol Chem.

[38] Goldenstein H, Levy NS, Ward J, Costacou T, Levy AP (2018). Haptoglobin genotype is a determinant of hemoglobin adducts and vitamin E content in HDL. J Diabetes Res.

[39] Rosú SA, Rimoldi OJ, Prieto ED, Curto LM, Delfino JM, Ramella NA, Tricerri MA (2015). Amyloidogenic propensity of a natural variant of human apolipoprotein AI: stability and interaction with ligands. PLoS One.

[40] Zannis VI, Chroni A, Krieger M (2006). Role of apoA-I, ABCA1, LCAT, and SR-BI in the biogenesis of HDL. J Mol Med.

[41] Daniil G, Zannis VI, Chroni A (2013). Effect of apoA-I mutations in the capacity of reconstituted HDL to promote ABCG1-mediated cholesterol efflux. PLoS ONE.

[42] Manthei KA, Yang SM, Baljinnyam B, Chang L, Glukhova A, Yuan W (2018). Molecular basis for activation of lecithin:cholesterol acyltransferase by a compound that increases HDL cholesterol. Elife.

[43] Cho KH, Durbin DM, Jonas A (2001). Role of individual amino acids of apolipoprotein A-I in the activation of lecithin: cholesterol acyltransferase and in HDL rearrangements. J Lipid Res.

[44] Yin K, Liao DF, Tang CK (2010). ATP-binding membrane cassette transporter A1 (ABCA1): a possible link between inflammation and reverse cholesterol transport. Mol Med.

[45] Hathaway CA, Heistad DD, Piegors DJ, Miller FJ (2002). Regression of atherosclerosis in monkeys reduces vascular superoxide levels. Circ Res.

[46] Levine GN, Keaney JFJ, Vita JA (1995). Cholesterol reduction in cardiovascular disease: clinical benefits and possible mechanisms. N Engl J Med.

